# The impact of relationship between tumor volume and radiation dose on pain relief: are higher doses needed for larger tumors?

**DOI:** 10.1093/jrr/rraf039

**Published:** 2025-07-21

**Authors:** Kohsei Yamaguchi, Tetsuo Saito, Tomohiko Matsuyama, Yoshiyuki Fukugawa, Takahiro Watakabe, Shigeo Yamada, Natsuo Oya

**Affiliations:** Department of Radiation Oncology, Ariake Medical Center 2600, Arao, Kumamoto 864-0041, Japan; Division of Integrative Medical Oncology, Saiseikai Kumamoto Hospital 5-3-1, Chikami Minami-ku, Kumamoto 861-4193, Japan; Department of Radiation Oncology, Kumamoto University Hospital 1-1-1, Honjo Chuo-ku, Kumamoto 860-8556, Japan; Department of Radiation Oncology, Kumamoto University Hospital 1-1-1, Honjo Chuo-ku, Kumamoto 860-8556, Japan; Department of Radiation Oncology, Kumamoto University Hospital 1-1-1, Honjo Chuo-ku, Kumamoto 860-8556, Japan; Department of Radiation Oncology, Kumamoto University Hospital 1-1-1, Honjo Chuo-ku, Kumamoto 860-8556, Japan; Department of Radiation Oncology, Kumamoto University Hospital 1-1-1, Honjo Chuo-ku, Kumamoto 860-8556, Japan

**Keywords:** palliation, palliative radiotherapy, pain relief, dose–volume effects

## Abstract

The influences of tumor volume and total radiation dose on pain relief outcomes have not been fully investigated. We investigated potential correlations between gross tumor volume (GTV), biologically effective dose (BED) and pain relief in patients receiving radiation therapy (RT) for painful tumors. As a secondary analysis of a three-center prospective observational study of 302 patients who received RT for painful tumors, patients treated at an academic hospital were analyzed. We used the Brief Pain Inventory short form to evaluate pain intensity and interference in patients’ lives. We collected the Brief Pain Inventory and analgesic data at baseline and 1, 2, 3, 6, 9 and 12 months after the start of RT. Pain responses were assessed using the International Consensus Endpoint. The Fine and Gray models were used for univariable and multivariable analyses, to estimate the impact of clinical factors on pain response and pain progression. In total, 153 (59%) of the 258 patients experienced a pain response, and 45 (17%) patients experienced pain progression. In the univariable and multivariable analyses, GTV and BED did not significantly associate with pain response or pain progression. Furthermore, no significant interaction between GTV and BED was reported in terms of pain response or pain progression after adjusting for covariates. The impact of BED on pain response and pain progression did not vary according to the GTV.

## INTRODUCTION

Radiation therapy (RT) plays a pivotal role in the management of cancer-related pain by providing effective palliation and improved quality of life for patients with painful tumors [1, 2]. Currently, a wide variety of radiation dose fractionation schedules are employed for pain relief [3]. Several studies have demonstrated that short courses of RT are equivalent to longer courses of RT in terms of pain relief [4–6]. In other words, no significant correlation between pain relief and total radiation dose has been found [7, 8].

In contrast, the total radiation dose is critical for local control because tumor volume, which correlates with the number of clonogenic tumor cells, influences local control [9, 10]. Previous studies have explored the effect of tumor volume and total radiation dose on local control in various types of cancer [11–16]. In these investigations, tumor volume and total radiation dose were found to be important determinants of treatment response and disease progression. However, the influences of tumor volume and total radiation dose on pain relief outcomes have not been fully investigated.

Based on prior tumor control findings [12, 16], patients with smaller tumors may experience improved pain relief with a lower total radiation dose and the impact of tumor volume might vary with the total radiation dose. That is, a higher total radiation dose might overcome the negative effect of a larger tumor volume. We hypothesized that there may be an interaction between the total radiation dose and tumor volume in terms of pain relief. In other words, to improve pain caused by larger tumors, a higher total radiation dose may be necessary. In this study, we investigated potential correlations among tumor volume, total radiation dose and pain relief in patients who received RT for painful tumors.

## MATERIALS AND METHODS

### Patients and study design

We performed a secondary analysis of a previously published prospective, three-center (one academic and two non-academic hospitals) observational study. In the primary study, 302 patients were scheduled to receive RT for painful tumors [7]. We sought to identify the predictors of pain response after RT for painful tumors. Among these patients, those treated in an academic hospital were analyzed in the present study. In the original study, radiation dose fractionation and target volumes were determined at the discretion of the radiation oncologists [7]. The present study was approved by the institutional review boards of the participating centers. Written informed consent was obtained from all the patients enrolled in the primary study.

### Evaluation

The patients were evaluated as previously described [7]. The Brief Pain Inventory (BPI) short form was used to evaluate pain intensity and interference in patients’ lives on an 11-point scale (0–10) [17]. A higher BPI score indicates more intense pain, greater disability and poorer well-being. Patients reported the worst pain they experienced (in terms of the index pain caused by the irradiated tumor [18]) within the previous 3 days. The BPI assesses pain interference using seven subscales: general activity, mood, walking ability, normal work, relations with other people, sleep and enjoyment of life [17]. BPI pain interference is typically scored as the mean of seven interference items, which can be used if more than 50%, or four out of seven items, are present in a given administration [19].

We collected BPI and analgesic data at baseline and at 1, 2, 3, 6, 9 and 12 months after the start of RT. The pain response was assessed and compared with baseline using the International Consensus Endpoint for clinical trials of bone metastases [20]. A complete response was defined as an index pain score of 0 with no increase in the daily oral morphine equivalent dose (OMED) [20]. A partial response was defined as a reduction in pain score of ≥2, without an increase in OMED or analgesic use reduction by ≥25% with no increase in pain score. A pain progression was defined as an increase in the index pain score of ≥2 without reduced OMED or an increase of ≥25% in the OMED without a decrease in pain score. An indeterminate response was defined as any response that did not qualify as a complete response, partial response or pain progression. Patients who received RT for painful tumors were assessed to determine the presence of a ‘pain response’, up to the third month. The pain responses included complete and partial responses. The patients were also assessed for the time to the appearance of pain progression through the 12th month.

In addition to index pain (pain caused by the irradiated tumor), non-index pain was assessed [18]. At baseline and follow-up, treating radiation oncologists prospectively evaluated the causes of non-index pain and whether the patients experienced pain other than index pain.

In the present study, gross tumor volume (GTV) was used to assess the tumor volume. If the GTV was defined at the time of treatment, it was used as is; otherwise, one radiation oncologist (KY) contoured and defined it.

To evaluate the radiation dose effect among various radiation dose fractionation schedules, the RT dose was converted to a biologically effective dose (BED) using an alpha/beta ratio of 10.

### Statistical analysis

Fine and Gray models were used for univariable and multivariable analyses, to estimate the impact of clinical factors on pain response and pain progression [21]. Our primary aim was to test the interaction between the GTV and BED in relation to pain response or pain progression. Namely, we investigated whether a greater BED was beneficial, especially when the irradiated tumors were large. Death without pain response or pain progression was treated as a competing risk. Fifteen potential predictors were selected as independent variables: GTV, BED, age, sex, Eastern Cooperative Oncology Group performance status, bone metastasis, hematologic tumor, interval from first tumor diagnosis to RT, pain duration, worst pain score at baseline, mean pain interference score at baseline, neuropathic component of index pain, non-index pain of malignant or unknown origin, opioid analgesic use and concurrent systemic therapy. The variance inflation factor was used to detect multicollinearity between independent variables. The cumulative incidence of pain response or pain progression and death without pain response or pain progression was calculated [22]. The GTV and BED were dichotomized based on their median values. All the *P* values were two-sided, and *P* values < 0.05 were considered significant. Statistical analyses were performed using R version 4.4.2.

## RESULTS

### Patients and treatment characteristics

The characteristics of the 258 patients who underwent RT between July 2013 and September 2017 and were included in this study are shown in Table 1. The primary sites of the solid tumors (*n* = 223) were the lungs (*n* = 62), gastrointestinal system (*n* = 58), gynecological system (*n* = 46), head and neck (*n* = 17), urogenital system (*n* = 12), breasts (*n* = 10), skin (*n* = 5) and others (*n* = 13). Of the 223 patients with solid tumors, 63 received RT for the primary lesion. The most commonly used RT schedule was 3 × 10 Gy, which was administered to 78 patients. Thirty patients received a single-fraction regimen (8 × 1 Gy), and nine patients did not complete the planned RT.

**Table 1:** Baseline patient characteristics (*n* = 258)

Characteristic	No.	%
Age, years		
Median	66	
Range	21–91	
Sex		
Female	117	45
Male	141	55
ECOG performance status		
0	56	22
1	99	38
2	66	26
3	35	14
4	2	1
Irradiated site		
Head and neck	29	11
Thorax	68	26
Abdomen and pelvis	129	50
Extremity	32	12
Total radiation dose, Gy		
Median	30	
Range	6–70.4	
≤10	32	12
10–20	30	12
20–30	89	35
30–40	28	11
>40	79	31
Biologically effective dose (alpha/beta = 10), Gy		
Median	39	
Range	7.8–85.9	
≤39 Gy (low-dose group)	151	59
>39 Gy (high-dose group)	107	41
Bone metastasis		
No	148	57
Yes	110	43
Hematologic tumor		
No	223	86
Yes	35	14
Gross tumor volume, cm^3^		
Median	86	
Range	3–1408	
≤86 cm^3^ (small-tumor group)	129	50
>86 cm^3^ (large-tumor group)	129	50
Interval from first tumor diagnosis to radiotherapy, months		
Median	7	
Range	0–557	
Pain duration, months		
Median	2	
Range	0.3–48.0	
Worst pain score at baseline		
0–2	7	3
3–4	56	22
5–7	87	34
8–10	108	42
Mean pain interference score at baseline		
Median	4.4	
Range	0–10	
Neuropathic component of index pain		
No	182	71
Yes	76	29
Non-index pain of malignant or unknown origin at baseline		
No	227	88
Yes	31	12
Opioid analgesic use at baseline		
No	119	46
Yes	139	54
Concurrent systemic therapy		
No	104	40
Yes	146	57
Data not available	8	3

Abbreviation: ECOG, Eastern Cooperative Oncology Group.

**Fig. 1:**
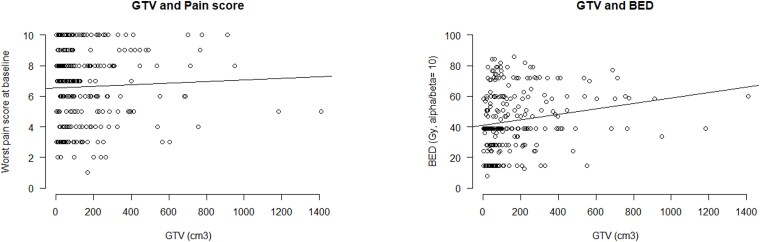
Scatter plot and regression line. GTV, gross tumor volume; BED, biologically effective dose.

The GTV was defined at the time of treatment in 237 patients. In 21 patients, the GTV was contoured by a radiation oncologist (KY). Figure 1 shows the scatter plots and linear regression lines that show the relationships between the GTV and pain score and between the GTV and BED in all 258 patients. The GTV and BED showed a slightly positive correlation (*r* = 0.035, *P* = 0.002), whereas the pain score did not correlate with the GTV (*r* = 0.002, *P* = 0.483).

#### Pain response, pain progression and analgesic use

In total, 153 (59%) of the 258 patients experienced a pain response (complete or partial response). The pain response rates for the evaluable patients at the 1-, 2- and 3-month follow-ups were 53%, 58% and 61%, respectively. The intention-to-treat pain response rates for all 258 patients were 47%, 46% and 38% at 1-, 2- and 3-month follow-ups, respectively. Forty-five patients (17%) experienced a pain progression. At baseline and at 1, 2 and 3 months of follow-up, the median index pain scores were 7, 3, 1.5 and 1, respectively. The median daily OMED values at baseline and at 1, 2 and 3 months of follow-up were 14, 15, 0 and 0 mg, respectively.

#### Relationships between pain response and patient characteristics and between pain progression and patient characteristics

Although there were eight missing values for concurrent systemic therapy (Table 1), complete case analyses were performed because the amount of missing data was minimal. A total of 21 patients (8%) died without experiencing a pain response, and 133 patients (52%) died without experiencing pain progression; these patients were considered to have experienced a competing event. Table 2 shows the analysis used to evaluate the potential predictors of pain response and pain progression using the Fine and Gray models. The univariable analysis suggested that sex (hazard ratio [HR] 0.72; 95% confidence interval [CI] 0.53–0.97; *P* = 0.034), pain duration (HR 0.93; 95% CI 0.89–0.98; *P* = 0.0053), non-index pain of malignant or unknown origin at baseline (HR 0.43; 95% CI 0.23–0.79; *P* = 0.0069) and opioid analgesic use at baseline (HR 0.70; 95% CI 0.51–0.95; *P* = 0.022) were significantly associated with pain response. The findings from the univariable analysis indicated significant associations between pain progression and variables such as worst pain score at baseline (HR 0.80; 95% CI 0.70–0.90; *P* = 0.00048) and non-index pain of malignant or unknown origin at baseline (HR 2.06; 95% CI 1.00–4.26; *P* = 0.050).

**Table 2:** Univariable Fine–Gray models for pain response and pain progression (*n* = 258)

	Pain response			Pain progression		
Variable	Subdistribution HR	95% CI	*P*	Subdistribution HR	95% CI	*P*
GTV						
≤86 cm^3^ (small-tumor group)	1.00 (reference)			1.00 (reference)		
>86 cm^3^ (large-tumor group)	1.17	0.86–1.59	0.33	0.66	0.36–1.20	0.16
BED (alpha/beta = 10)						
≤39 Gy (low-dose group)	1.00 (reference)			1.00 (reference)		
>39 Gy (high-dose group)	1.26	0.93–1.71	0.14	0.99	0.55–1.80	0.97
						
Age, years (continuous)	0.99	0.98–1.01	0.37	1.01	0.99–1.03	0.29
Sex						
Female	1.00 (reference)			1.00 (reference)		
Male	0.72	0.53–0.97	0.034	0.98	0.55–1.75	0.95
ECOG performance status						
0, 1	1.00 (reference)			1.00 (reference)		
2–4	1.13	0.82–1.58	0.45	0.80	0.59–1.07	0.13
Bone metastasis						
No	1.00 (reference)			1.00 (reference)		
Yes	0.80	0.58–1.10	0.16	1.55	0.87–2.80	0.14
Hematologic tumor						
No	1.00 (reference)			1.00 (reference)		
Yes	1.48	0.94–2.32	0.088	0.58	0.21–1.60	0.29
Interval from first tumor diagnosis to radiotherapy, months (continuous)	1.00	1.00–1.00	0.76	1.00	1.00–1.00	0.31
Pain duration, months (continuous)	0.93	0.89–0.98	0.005	1.04	1.00–1.08	0.086
Worst pain score at baseline (continuous)	1.04	0.98–1.11	0.20	0.80	0.70–0.90	<0.001
Mean pain interference score at baseline (continuous)	1.00	0.95–1.06	0.94	0.95	0.86–1.05	0.33
Neuropathic component of index pain						
No	1.00 (reference)			1.00 (reference)		
Yes	1.31	0.95–1.80	0.096	0.89	0.46–1.73	0.73
Non-index pain of malignant or unknown origin at baseline						
No	1.00 (reference)			1.00 (reference)		
Yes	0.43	0.23–0.79	0.007	2.06	1.00–4.26	0.050
Opioid analgesic use at baseline						
No	1.00 (reference)			1.00 (reference)		
Yes	0.70	0.51–0.95	0.022	0.86	0.48–1.53	0.60
Concurrent systemic therapy						
No	1.00 (reference)			1.00 (reference)		
Yes	0.88	0.64–1.21	0.42	1.21	0.66–2.24	0.54

Abbreviations: GTV, gross tumor volume; BED, biologically effective dose; ECOG, Eastern Cooperative Oncology Group; HR, hazard ratio; CI, confidence interval.

#### The interaction between tumor volume (GTV) and radiation dose (BED) on pain response and pain progression

To evaluate whether there was a significant interaction between tumor volume (GTV) and radiation dose (BED), the product of GTV and BED (GTV × BED) was included as a covariate in the Fine and Gray models. Table 3 shows the results of the multivariable analysis, with and without significant covariates in the univariable analysis. No significant interaction was observed between the GTV and BED in terms of pain response or pain progression; in other words, the impact of BED on pain response and pain progression did not vary according to the GTV.

**Table 3:** Multivariable Fine–Gray models for pain response and pain progression (*n* = 258)

	Without covariates			With covariates^a^		
Variable	Subdistribution HR	95% CI	*P*	Subdistribution HR	95% CI	*P*
Pain response						
GTV						
≤86 cm^3^(small-tumor group)	1.00 (reference)			1.00 (reference)		
>86 cm^3^(large-tumor group)	1.00	0.62–1.63	0.99	1.10	0.67–1.82	0.70
BED (alpha/beta = 10)						
≤39 Gy (low-dose group)	1.00 (reference)			1.00 (reference)		
>39 Gy (high-dose group)	1.10	0.68–1.76	0.70	1.13	0.70–1.84	0.61
GTV × BED	1.23	0.64–2.37	0.53	0.95	0.48–1.88	0.88
Sex						
Female				1.00 (reference)		
Male				0.78	0.56–1.07	0.12
Pain duration, months (continuous)				0.93	0.89–0.98	0.005
Non-index pain of malignant or unknown origin at baseline						
No				1.00 (reference)		
Yes				0.46	0.24–0.88	0.018
Opioid analgesic use at baseline						
No				1.00 (reference)		
Yes				0.76	0.55–1.05	0.10
Pain progression						
GTV						
≤86 cm^3^(small-tumor group)	1.00 (reference)			1.00 (reference)		
>86 cm^3^(large-tumor group)	0.94	0.43–2.06	0.88	0.95	0.45–2.02	0.90
BED (alpha/beta = 10)						
≤39 Gy (low-dose group)	1.00 (reference)			1.00 (reference)		
>39 Gy (high-dose group)	1.58	0.74–3.39	0.24	1.21	0.56–2.62	0.62
GTV × BED	0.42	0.13–1.40	0.16	0.47	0.14–1.57	0.22
Worst pain score at baseline (continuous)				0.79	0.68–0.90	<0.001
Non-index pain of malignant or unknown origin at baseline						
No				1.00 (reference)		
Yes				1.94	0.94–4.02	0.075

Abbreviations: GTV, gross tumor volume; BED, biologically effective dose; HR, hazard ratio; CI, confidence interval.

Note. GTV, BED and their interaction term (i.e. GTV × BED) were included in all the models.

^a^Covariates with a *P* value < 0.05 at univariable analyses (Table 2) were included in the analysis.

**Fig. 2:**
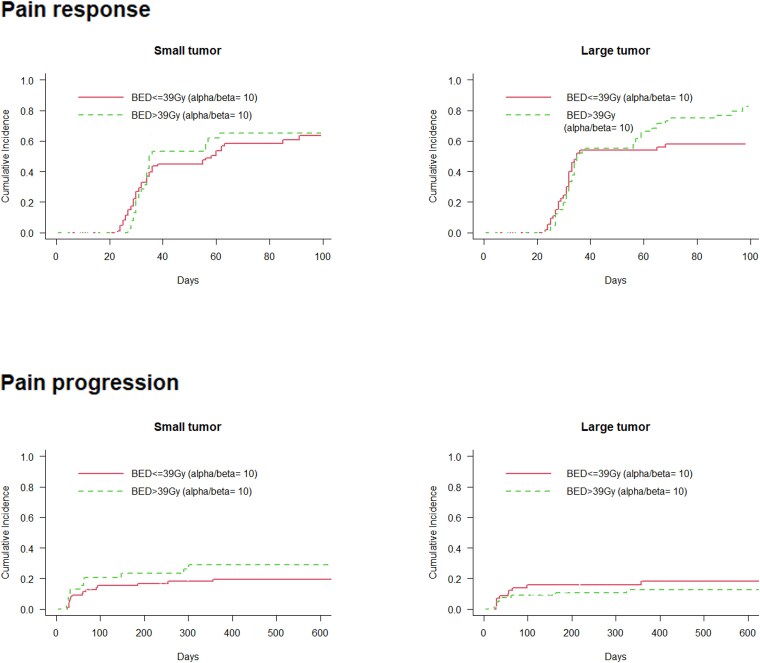
Cumulative incidence of pain response and pain progression. BED, biologically effective dose.

To evaluate the effect of BED in different GTV groups, patients were separated based on the median value of BED (≤39 Gy vs >39 Gy) and GTV (≤86 cm^3^, small-tumor group, vs >86 cm^3^, large-tumor group). Figure 2 shows the effects of the BED on the cumulative incidence of pain response and pain progression in the GTV group. BED was not significantly associated with the cumulative incidence of pain response in the small- (*P* = 0.73) and large-tumor groups (*P* = 0.18). No significant association was observed between BED and the cumulative incidence of pain progression within the small- (*P* = 0.17) and large-tumor groups (*P* = 0.46).

## DISCUSSION

This study investigated the interactions among tumor volume, total radiation dose and pain relief in patients receiving RT for painful tumors. To the best of our knowledge, this is the first study to analyze whether the effect of the total radiation dose on pain relief varies with tumor volume. Although tumor volume has been identified as a significant determinant of local control in previous studies [11, 13, 15, 16], the impact of tumor volume on pain relief outcomes has not been thoroughly investigated before the present study. Notably, the interaction term (GTV × BED) was not significantly associated with the pain response or pain progression. This finding suggests that GTV and BED do not interact significantly in terms of pain response (response to RT) or pain progression (duration of response to RT). Thus, higher radiation doses do not appear necessary to improve pain relief in patients with larger tumors.

Prior evidence supports the absence of a significant association between radiation dose and pain response [4]. Additionally, our findings indicated that a dose–response relationship does not exist, even when tumor volume is considered. The absence of a dose–response relationship in terms of pain control, despite the existence of a dose–response relationship in terms of tumor control, may be explained by the mechanism of pain palliation by RT. Although the exact mechanism underlying radiation-induced pain relief remains unknown, it is assumed to be multifaceted. Certain features of the response such as diminishing pain after a few sessions of RT suggest that tumor shrinkage is unlikely to account for the early period of pain relief. Inflammatory cells and osteoclasts may play important roles in pain relief following RT [23]. The reduction in inflammatory cells by ionizing radiation may inhibit the release of chemical pain mediators [24]. Urinary markers of osteoclastic activity correlate with pain relief after RT [25].

Although our analyses on pain progression did not provide evidence supporting the use of higher radiation doses for long-term symptom palliation, these findings should not be extrapolated to clinical situations where sustained tumor control is required, such as RT for oligometastatic disease. In certain patients, local tumor control may have clinical significance, particularly in the modern era of improved systemic therapies and prolonged survival even among those with distant metastases.

This study had some limitations. First, it relied on a secondary prospective analysis of data from a previously published observational study, and our results should be confirmed in future studies. Second, the subgroup analysis used data from a single academic institution (originally one of three sites) due to changes in author affiliation that made further contouring unfeasible. While this may introduce a potential bias, the analyzed cohort still comprised 85% of the original 302 patients, most of whom were from the academic site. Moreover, during enrollment, the same physician (the study coordinator) was involved in the original prospective study across all the sites, probably contributing to consistent clinical practice. Thus, although this is a limitation, it is unlikely to significantly affect the findings. Third, this study investigated RT for painful tumors in an academic hospital, which may limit the generalizability of the findings to other settings or treatment modalities. Fourth, there is no solid evidence supporting the use of any specific alpha/beta ratio when calculating BED for analyzing the effect of pain palliation after RT. In this study, we used the alpha/beta ratio of 10, which has conventionally been employed in such analyses [26, 27]. Notably, the strengths of this study include its prospective design, use of a validated pain assessment tool (BPI and International Consensus Endpoint) and inclusion of various potential predictors of pain response and pain progression as covariates in the multivariable analyses.

In summary, there was no significant interaction between GTV and BED in terms of pain response or pain progression; that is, the radiation dose was not associated with pain relief, regardless of tumor volume. Therefore, our study found no evidence to support the use of higher radiation doses for patients with larger tumors.

## PRESENTATION AT A CONFERENCE

This study was presented at the 35th Annual Meeting of the Japanese Society for Radiation Oncology.
